# Relationship between Hypothyroidism and Endometrial Cancer

**DOI:** 10.14336/AD.2018.0224

**Published:** 2019-02-01

**Authors:** Yiqin Wang, Rong Zhou, Jianliu Wang

**Affiliations:** Department of Obstetrics and Gynecology, Peking University People’s Hospital, Beijing, China

**Keywords:** Hypothyroidism, Endometrial cancer, Sex hormone, Metabolic syndrome, Fertility

## Abstract

Thyroid dysfunction is involved in several types of carcinoma. Hypothyroidism is one of the most common medical morbidities among patients with endometrial cancer; however, the related mechanism is unclear. Among the risk factors related to endometrial cancer, hypothyroidism interacts with metabolic syndrome, polycystic ovarian syndrome and infertility or directly acts on the endometrium itself, which may influence the development and progression of endometrial cancer. We summarize recent studies on the relationship between hypothyroidism and endometrial cancer and its risk factors to provide references for basic research as well as for clinical treatment and prognostic evaluation.

Review

Endometrial cancer (EC) is one of the most common gynecological malignancies. Obesity, diabetes mellitus, hypertension, a continuous estrogen supply unopposed by progesterone, infertility, nulliparity, and tamoxifen use are widely accepted to be in involved in the pathogenesis of EC. Clinicians do not usually test the thyroid function of patients with EC, but, interestingly, hypothyroidism has been reported to be a common complication of EC, leading us to consider whether hypothyroidism is also a risk factor for EC. Currently, evidence on the direct relationship between hypothyroidism and EC is unavailable. However, studies have investigated the influence of thyroid function on the above risk factors related to EC. In this review, we will summarize the research on this issue.

## Hypothyroidism

Hypothyroidism is a type of reduced metabolic syndrome caused by inadequate synthesis and secretion of thyroid hormone (TH) or insufficient utilization by tissues. Primary hypothyroidism is due to thyroid gland dysfunction and constitutes approximately 99% of all cases of hypothyroidism [1]. The prevalence of primary hypothyroidism is 4-10% in healthy individuals, and it is higher among women and the elderly. Additionally, the incidence increases with age, reaching 20% in women older than 60 years. Measures of thyroid-stimulating hormone (TSH), total thyroxine (TT4), and free thyroxine (FT4) are the first-line diagnostic tests for hypothyroidism. TSH is the most sensitive indicator of thyroid dysfunction. Its reference range is 0.3-4.8 mIU/l [2]. Common clinical features associated with hypothyroidism include chills, fatigue, somnolence, hypomnesis, weight gain, menstrual disorders and infertility. The goal of treatments for primary hypothyroidism is to remove symptoms and signs, and maintain a normal range of serum TSH, TT4 and FT4 levels. Levothyroxine supplementation is the main treatment for hypothyroidism. Subclinical hypothyroidism refers to increased TSH levels, while TT4 and FT4 levels are within the normal range. The prevalence rates of hypothyroidism and subclinical hypothyroidism are 0.3% and 4.3%, respectively [2].

## Epidemiology of Hypothyroidism in EC

Thyroid disorders are associated with the prognosis of cancer. Hypothyroidism plays different roles in different types of cancer. High serum TSH levels have been reported to improve the outcomes of treatments for head-and-neck cancer, glioma, and breast cancer, but these levels are related to poor outcomes in renal cell cancer [3-6]. This inconsistency indicates differences in tumorigenesis between EC and other types of cancer and suggests that hypothyroidism might be involved in EC via another process. To date, few articles examining this relationship have been published.

Hypothyroidism is a common complication of EC. As shown in the 2001 study by Sharma et al. [7], EC might be related to hypothyroidism; of the 24 patients with associated medical disorders among 113 EC cases, the authors unexpectedly found that five patients had hypothyroidism, which might also be a risk factor associated with EC. According to Brinton et al. [8], EC is related to previous diagnoses of thyroid diseases (RR=1.52, 95% CI 1.17-1.98) and obesity (2.05, 1.40-3.00), but the data do not show which specific types of thyroid disease were more strongly linked to the risk. Siriwan et al. [9] examined 335 patients with EC and found that 220 (65.7%) had medical co-morbidities, of which two or more components of metabolic syndrome was the most common (72.3%). Thyroid dysfunction was the second most common co-morbidity and was observed in 8.2% of patients, whereas the incidence of hypothyroidism was 4.2%. Mine et al. [10] studied 135 patients with EC and found significantly elevated serum TSH concentrations compared to those in 135 healthy controls (2.63±1.09 versus 2.31±0.97 mIU/ml, P=0.012). In the study by Yurkovetsky et al. [11], patients with EC also presented higher serum TSH levels than the control group: 3.9±0.36 versus 2.4±0.65 ng/ml (P<0.0001). Besides, George’s [12] prospective study also found 8 of 161 women with endometrial hyperplasia complicated with hypothyroidism. Thus, patients with EC tend to have hypothyroidism, suggesting that thyroid function and hormones might be related to EC; this relationship should be explored in greater detail.

## Hypothyroidism and Clinicopathological Characteristics of EC

Hypothyroidism appears to be related to a poor prognosis for patients with EC. A multi-center trial undertaken by Seebacher et al. [13] in 2013 was the first to investigate the influence of TSH on the prognoses of patients with EC. By ranking 199 patients with EC according to the TSH level, an elevated TSH level was shown to be independently related to poor disease-specific survival in univariate and multivariate survival analyses (P=0.01 and P=0.03, respectively). Thus, serum TSH measurements might be used as an independent prognostic parameter for the survival of patients with EC to determine recurrence during EC follow-up. However, in this article [13], no associations were observed between elevated pre-therapeutic serum TSH levels and advanced FIGO tumor stage, high histological grades, unfavorable histological subtypes, older patient ages, or lifestyle factors, such as obesity, hypertension, or diabetes mellitus. Based on this finding, TSH might be associated with systemic processes that interact with carcinogenesis (e.g., hormonal imbalances or inflammation) rather than with local neoplastic transformation. However, the specific mechanism by which the serum TSH level influences EC remains unknown. Kang’s large cohort study of 1314 patients with EC reported an inconsistent result: a history of hypothyroidism or hyperthyroidism was not significantly associated with the risk of EC. Nevertheless, the article did not measure the thyroid hormone levels and only used self-reports of a history of thyroid disorder [14].

### Hypothyroidism and EC Risk Factors

According to the literature, the incidence of hypothyroidism in patients with EC is significantly elevated, and the serum TSH level before treatment is an independent risk factor for a poor prognosis of EC [13]. Therefore, studies determining the mechanism by which hypothyroidism is related to EC are necessary. Risk factors for EC include obesity, diabetes mellitus, postmenopausal estrogen replacement, ovarian dysfunction, infertility, nulliparity and tamoxifen use. Most of the risk factors for EC are explained within the framework of the unopposed estrogen hypothesis, which proposes that long-term exposure to estrogen increases the mitotic activity of endometrial cells and increases the numbers of DNA replication errors and somatic mutations, resulting in malignant phenotypes. In addition, due to the lack of differentiation mediated by progesterone, the endometrium then transforms to exhibit atypical hyperplasia and malignancies [15, 16]. Some researchers have investigated the relationship between hypothyroidism and the present known risk factors for EC. Hypothyroidism might influence the development of EC by interfering with the above risk factors, either indirectly or through other dependent mechanisms. A summary of our findings is presented in Table 1.

**Table 1:** Relationship between hypothyroidism and risk factors for EC.

Parameter	MS			PCOS	Infertility	Leptin
	Dyslipidemia	Obesity	Hypertension			
Relationship	MS ↑, TC ↑, LDL-C ↑, triglyceride ↑, HDL ↓ [17-19]	BMI ↑ [20]	BP ↑ [21, 22]	Ovarian volume and cyst formation ↑; PCOS ↑; CC resistance ↑[23-26].	Menstrual problems, breakthrough bleeding, anovulation, and infertility↑ [27, 28].	Leptin ↑
Possible mechanism	TH → SREBP-2 and LDL-receptors ↑ → cholesterol removal from the liver [29]; TH → lipoprotein lipase and hepatic lipase activity ↑ → serum TG and cholesterol↓ [30]; TSH → acting on TSH receptors in the hepatocyte membranes → HMGC ↑ → cholesterol synthesis in the liver ↑ [19]; TSH acts on preadipocyte differentiation and adipogenesis → lipolysis↑→ serum free fatty acid↑ [19].	-	T3 acts on arterial smooth muscle cells of blood vessels to cause vasodilation [31]. Hypothyroidism → abnormal sodium metabolism, the sympathetic nervous system activity ↑; glomerular filtration rate↓[32, 33].	Variant FBN3 →TGFβ and Treg ↓ → development of autoimmunity; anovulation → E/P → autoantibodies ↑; SHBG↓ →free sex hormones↑ [34-36].	Clearance of androstendione and estrone ↓ → aromatization, leading to free estrogen ↑; TRH ↑ →PRL ↑; GnRH pulse change → LH peak delays and luteum function disorders and coagulation defects [27, 37].	Actions on adipocytes → stimulate leptin secretion [38].

### Hypothyroidism and Metabolic Diseases

Elevated TSH levels have been reported to increase the incidence of metabolic syndrome (MS) [17]. TSH levels are significantly related to lifestyle factors, such as BMI, weight, waist circumference, metabolic diseases, including hypertension, and levels of low-density lipoprotein cholesterol (LDL-C), total cholesterol (TC) and high-density lipoprotein cholesterol (HDL-C) [17]. However, Seebacher [13] retrospectively investigated the relationships between pretreatment TSH levels and clinicopathological parameters in 199 patients with EC and found that TSH levels were not related to lifestyle factors, such as obesity (P=0.4), hypertension (P=0.9) or diabetes (P=0.5).

Dyslipidemia is the most commonly observed metabolic disease related to hypothyroidism. Patients with dyslipidemia constitute 1.4% to 13.3% of all patients with hypothyroidism [22]. Much research has been conducted on the relationship between hypothyroidism and dyslipidemia. According to the population study by Morris et al. [18], the odds ratios (OR) for hypothyroidism associated with high levels of TC and LDL-C were 8.0 and 5.3, respectively. As shown in a cross-sectional study by Xu et al. [19] on patients with coronary heart disease, the TSH level is positively correlated with the TC level (r=0.095, P=0.036), whereas FT3 and T4 levels are negatively correlated with the TC level (r=-0.125, P=0.003 and r=-0.190, P<0.001 respectively). Shinkov [17] conducted a cross-sectional study of 2153 euthyroid subjects and found that within normal limits, the prevalence of MS increased with elevated TSH levels, mostly appearing as an increase in dyslipidemia. Thus, MS was more prevalent in the group with the highest TSH level (34.9%) than in the group with the lowest level (27%) (P<0.001), as were low HDL-C levels (32% vs. 25%, P<0.001) and hypertriglyceridemia (26.8% vs. 20.4%, P=0.015). Moreover, treatments for hypothyroidism seem to improve lipid metabolism. According to some randomized controlled trials (RCT), T4 replacement exerts positive effects on dyslipidemia by improving TC and LDL-C levels [39, 40].

Some studies have proposed a mechanism for the abnormal lipid production induced by hypothyroidism. THs may increase the levels of sterol regulatory element-binding protein-2 (SREBP-2) and LDL-receptors, resulting in facilitation of cholesterol removal from the liver and a subsequent decrease in cholesterol levels [29]. Another mechanism suggests that an increase in lipoprotein lipase and hepatic lipase activities decreases serum TG and cholesterol levels [30]. Meanwhile, TSH upregulates the expression of HMGCR (the rate-limiting enzyme in cholesterol synthesis) by acting on TSH receptors in hepatocyte membranes and thereby promotes cholesterol synthesis in the liver and elevates cholesterol levels [19]. Furthermore, TSH directly affects preadipocyte differentiation and adipogenesis, stimulates lipolysis in cultured adipocytes and elevates serum free fatty acid levels in vivo [19]. However, the direct relationship between hypothyroidism and dyslipidemia requires further investigation.

Moreover, hypothyroidism might be related to obesity and hypertension. As shown in the study by Knudsen, BMI was positively correlated with the serum TSH level (P<0.001) and negatively correlated with the FT4 level (P<0.001). Compared with the group with a serum TSH level of 1.0-1.99 mIU/l, the group with a TSH level above 3.6 mIU/l had a 2.1-fold increased OR for obesity (BMI > 30 kg/m^2^) [20].

According to the cross-sectional population study by Liu et al. [21], patients with subclinical hypothyroidism had a higher blood pressure than patients with euthyroid function (41.3% and 25.6%, P<0.05), and after adjusting for age, smoking status, IR, and BMI, the conclusion remained consistent (OR 3.545, P=0.004). Luboshitzky [22] also reported a higher prevalence of hypertension in patients with subclinical hypothyroidism. The increase in systemic vascular resistance may be the main mechanism of hypertension in patients with clinical hypothyroidism. T3 directly acts on the arterial smooth muscle cells of blood vessels to cause vasodilation [31]. Hypothyroidism also leads to abnormal sodium metabolism, increased activity of the sympathetic nervous system, and a decrease in the glomerular filtration rate, among other alterations, which may be involved in the occurrence of hypertension [32, 33].

### Hypothyroidism and Infertility

Hypothyroidism is closely related to infertility. Hypothyroidism can cause changes in the levels of sex hormones and is involved in many reproductive diseases, including abnormal sex gland development, menstrual problems (mainly oligo- or amenorrhea), breakthrough bleeding, anovulation, and infertility [27, 28]. Krassas et al. [37] concluded that 23% of 171 patients with hypothyroidism had menstrual disorders (mainly presenting as amenorrhea) compared with a disorder rate of 8% in a control group of 214 subjects (P<0.05). LH/FSH and T4 are required for fertilization and blastocyst development. According to Ctamer [41] TSH levels significantly predict fertilization failure among women undergoing IVF. Also, Sordia et al. [51] found that mild high thyroid stimulating hormone levels (≥2.5 mU/L) may adversely affect the pregnancy rates in patients undergoing in vitro fertilization (p < 0.001). Moreover, a T4 treatment has been shown to normalize the reaction to GnRH, thus decreasing the menstrual disorder and increasing the rate of spontaneous pregnancy [42].

Nevertheless, hypothyroidism is more common in women with fertility problems. The risk of AIT in infertile women is 2.1-fold greater than in normal controls [37]. Arojoki [43] studied 299 infertile women and found that 4% displayed elevated TSH levels, and the incidence of clinical hypothyroidism was 3.3%. Among the women with elevated TSH levels, ovulation dysfunction constituted the largest proportion of disorders (6.3%). Bohnet [44] conducted a TRH test for 185 25-34-year-old infertile women, 11% of which displayed TSH hyperreactivity (>20 mIU/l) and were considered to have subclinical hypothyroidism. After T4 treatment, 11 of the 20 patients displayed a normalization of progesterone secretion, and 2 women got pregnant.

The mechanism of action of hypothyroidism on infertility is unknown. Some experts believe have observed decreased clearance of androstendione and estrone and an increase in aromatization that lead to high free estrogen levels, increased thyrotropin-releasing hormone (TRH) levels that lead to increased prolactin (PRL) levels, changes in the gonadotropin-releasing hormone (GnRH) pulse that cause luteinizing hormone (LH) peak delays and luteum function disorders, and coagulation defects in individuals with hypothyroidism; all of these changes eventually cause ovulation and fertility problems [27, 37]. Binita [52] investigated 160 women with primary infertility and found a slightly higher prevalence of hypothyroidism in the infertile group in comparison with that of the general population, also there was a positive correlation between serum TSH and prolactin levels in the infertile subjects.

Therefore, hypothyroidism has been reported to induce menstrual and fertility problems, which tend to complicate amenorrhea and ovulation dysfunctions that are in turn also risk factors for EC.

### Hypothyroidism and Leptin

Leptin is a circulating hormone secreted by adipose tissue. As a signaling factor in the central nervous system, it regulates energy homeostasis and neuroendocrine functions. Leptin might increase EC risk [45-47]. According to the meta-analysis by Wang [45], a high leptin level was an independent risk factor for EC (RR 1.59, 95% CI 1.27-1.98, P<0.001), and for every 5 ng/ml increase in the leptin level, the OR for EC increases by 1.10 (95% CI, 1.03-1.18, P=0.005). Another analysis of the relationships between leptin, its receptor expressed in EC and the clinical and pathological parameters showed that leptin and its receptor were overexpressed in patients with EC [46]. Their expression levels were related to EC malignancy, invasiveness and metastatic potential, as well as a poor prognosis (3-year survival rate). Moreover, leptin and its receptor are also positively correlated with the expression of estrogen receptors. In this manner, leptin and its receptor might be important risk factors to be considered in EC. Recently, the mechanism underlying the effect of leptin on EC has been revealed. A study by Zhou [47] proposed that leptin inhibits EC apoptosis in vitro partially by inducing the activation of the NIK/IKK pathway, and overexpression of leptin receptors may facilitate EC progression. As shown in the study by Liu [48]. leptin promotes the development and invasiveness of EC by regulating the levels of pre-angiogenesis and pre-inflammatory factors.

Although hypothyroidism has been shown to increase leptin levels, the physiological mechanisms for the TH-induced alterations in serum leptin levels have not been completely elucidated. Menendez et al. [38] determined whether TSH stimulated leptin secretion by directly acting on adipocytes. Omental adipose tissue was obtained from 34 patients during selected abdominal surgeries to test the direct effect of TSH on leptin secretion. TSH significantly increased leptin secretion from adipose tissue in vitro, whereas it had no such effects on PRL, adrenocorticotropic hormone (ACTH), follicle stimulating hormone (FSH) or LH. Thus, serum TSH may regulate pulsatile variations in leptin levels. Oge et al. [49] investigated the relationships between TH and leptin levels in patients with overt hypothyroidism and hyperthyroidism before and after treatment. The serum leptin levels of patients with hypothyroidism (28.4±4.1 ng/ml) were significantly higher than the controls (19.1±3.2 ng/ml) (P<0.01), whereas patients with hyperthyroidism had lower levels (10.7±1.2 ng/ml) (P<0.01). In patients with hypothyroidism, thyroxin treatment significantly decreased serum leptin levels to 20.6±2.1 ng/ml (P<0.05). Therefore, serum leptin levels may be affected by thyroid disorders. In a study of 800 obese individuals by Betry, TSH and leptin levels maintained a significant association that was independent of the BMI. Further studies are needed to determine whether hypothyroidism promotes EC by increasing leptin levels.

## TSH Acts on the Endometrium

Some experts believe that TSH might act directly on the endometrium. Animal experiments have detected the expression of receptors for TRH, TSH and thyroxine in the monkey uterus and shown that their long-term levels are by sex hormones [18]. Lusine et al. [50] were the first to confirm that TR, TSH receptors (TSHRs), and iodothyronine deiodinase (DIO) were distributed in the endometrium in healthy women. Furthermore, TH levels were significantly elevated in cultivated Ishikawa cells stimulated with TH. The appearance of THs in the supernatant of endometrial interstitial cells and Ishikawa cells indicated that they are secreted by endometrial cells. After stimulation with TSH, TH secretion increased, indicating that active TSH receptors are present in endometrial cells, and bind to TSH to induce TH secretion independent of the hypothalamic-pituitary-thyroid axis. Furthermore, THs might bind to the TRs on the endometrial surface and then act partially via the paracrine pathway. The existence of DIO in the endometrium indicates that a T4 to T3 transition occurs via TRs [50]. Thus, TSH seems to act directly on the endometrium, but the specific mechanism remains to be determined.

## Conclusions

Based on this review of the available experimental and clinical data, hypothyroidism is not an uncommon complication of EC, and an increased pretreatment serum TSH level is related to a poor prognosis for patients with EC. Hypothyroidism exhibits an intimate relationship with the many risk factors for EC, including metabolic syndrome, PCOS, and infertility. Hence, hypothyroidism exhibits a complicated relationship with EC, perhaps by promoting the occurrence and development of EC by indirectly interfering with these risk factors or by directly acting on thyroid-related receptors and influencing the disease in unknown ways. Studies examining the relationship between thyroid function and EC will provide new insights into the mechanism of EC. Thyroid function and TSH levels may become tumor markers to evaluate the prognosis and facilitate follow-up monitoring of patients with EC.
